# Immunomodulatory actions of tonifying polysaccharides: pharmacological effects, mechanisms and therapeutic applications

**DOI:** 10.3389/fimmu.2025.1640679

**Published:** 2025-07-22

**Authors:** Jin-Yu Li, Chang Yi, Meng-Qin Zhu, Yan-Feng Yuan, Guang Chen, Ning-Ning Qiu, Lei Shen, Li-Ya Song, Wen-Long Liu, Xi-Li Zhang

**Affiliations:** ^1^ Pharmacy School, Hunan University of Chinese Medicine, Changsha, China; ^2^ Hunan Provincial Key Laboratory of Druggability and Preparation Modification of Traditional Chinese Medicine, Changsha, China; ^3^ Hengyang Traditional Chinese Medicine Hospital, Hengyang, China

**Keywords:** Tonifying polysaccharides, immunomodulation, pharmacological effects, mechanisms, therapeutic actions

## Abstract

Tonifying polysaccharides, as a class of natural medicines, have attracted considerable interest due to their low toxicity, high safety profile, and excellent stability. Investigations have highlighted the significant impact of polysaccharides derived from Chinese medicinal herbs on the modulation of immune responses, significantly enhancing the functionality of immune organs such as the spleen, thymus, bone marrow, and intestines, stimulating immune cell proliferation and activation of T and B lymphocytes, macrophages, natural killer cells, and dendritic cells, and regulating the secretion and release of immune factors, thereby enhancing overall immune function. Tonifying polysaccharides, such as those found in medicinal herbs like ginseng and Astragalus, have demonstrated significant therapeutic effects in treating immune-mediated diseases, including anti-tumor, anti-autoimmune, and anti-viral activities. Despite the extensive literature published on the pharmacological effects, mechanisms, and therapeutic applications of Tonifying polysaccharides, there remains a lack of systematic organization and summarization. This review summarizes recent research findings on Tonifying polysaccharides within the field of immunomodulation. The research explores the immunomodulatory mechanisms and therapeutic applications of polysaccharides, clarifying their roles in enhancing immune function and their potential in clinical treatments, and aims to establish a robust theoretical framework and scientific foundation for the investigation and practical application of Tonifying polysaccharides, drawing on the extensive research on their structural complexity, biological activities, and clinical applications, thereby promoting their development and application as immune-enhancing agents in traditional Chinese medicine and as vaccine adjuvants

## 1 Introduction

Immunity is a series of biological processes through which the organism recognizes self and non-self entities and mounts responses to maintain internal homeostasis and defend against microbial attacks, such as bacteria, viruses, and fungi. Immune system dysfunction can compromise the body’s disease resistance and may lead to the development of inflammation, diabetes, tumors, and a wide range of other diseases (1). Previous clinical and related studies have demonstrated that the primary risk factors for immune dysregulation are multidimensional, including genetic mutations, environmental pollution, chronic stress, sleep disorders, and drug effects (2). Currently, a wide variety of immunomodulatory drugs are available clinically, including chemically synthesized drugs and biological agents. However, these drugs generally exhibit limitations, such as single composition, limited target points, and a propensity to cause adverse reactions, including liver and kidney damage and metabolic disorders. Therefore, the development of novel immunomodulatory agents has emerged as a pressing scientific challenge.

Studies have demonstrated that natural medicines offer substantial advantages in enhancing therapeutic efficacy and reducing the incidence of adverse reactions (3). Polysaccharides, as a category of natural high molecular weight polymers, are prevalent in plants, animals, and microbial species, possess a complex and diverse structure composed of multiple monosaccharide molecules condensed through glycosidic bonding, and are characterized by low toxicity, high safety, strong stability and rich biological activity (4, 5). From a chemical structural perspective, the relative molecular mass of polysaccharides ranges from tens of thousands to millions and is typically composed of various monosaccharides, such as glucose, galactose, mannose, and arabinose, in specific proportions and bonding configurations. These monosaccharides are linked via α-glycosidic bonds or β-glycosidic bonds, forming linear or branched polysaccharide chains. The immunomodulatory activity of polysaccharides is closely related to the types and proportions of their constituent monosaccharides, the types of glycosidic bonds, and their molecular weight (6). In addition, the immunomodulatory effects of tonifying polysaccharides are not restricted to a solitary receptor or a discrete signaling cascade, but rather exhibit characteristics of multi-level, multi-pathway, and bi-directional target regulation, thereby modulating the body’s immune capacity (7). For example, the mechanism by which low-molecular-weight heteropolysaccharides derived from the fruiting bodies of *Ganoderma leucocontextum* (GLP-1) activate macrophages is closely related to three major signaling pathways: mitogen-activated protein kinases (MAPKs), phosphoinositide-3 kinase/protein kinase B (PI3K/Akt), and nuclear factor κB (NF-κB) (8). Tonifying polysaccharides primarily originate from plant-based sources (e.g., ginseng polysaccharide (9), *Astragalus* polysaccharide (10, 11), Radix astragali polysaccharides (12), *Lycium barbarum* polysaccharides (13), and *Polygonatum sibiricum* polysaccharides (14), fungal and algal sources (e.g., *Ganoderma lucidum* polysaccharide (15, 16), *Lentinan* (17, 18), and animal-based sources (e.g., Sika deer antler (19), honey bees (20). They play a crucial role in treating various deficiency syndromes, enhancing organ function, improving cellular and humoral immunity, and modulating immune function. Studies have demonstrated that tonifying polysaccharides possess natural immune-modulating properties and exhibit broad pharmacological activities, including antioxidant, antitumor, lipid-lowering, and antiviral effects. Therefore, tonifying polysaccharides, as safe, stable, and effective immunomodulatory agents, exhibit significant promise for treating diseases related to the immune system and have emerged as a current research hotspot.

In this analysis, we systematically synthesize the recent developments in tonifying polysaccharides within the domain of immunomodulation, discussing their effects on immune organs, immune cells, and mechanisms of action, and outlining their therapeutic potential in immune-related diseases. Tonifying polysaccharides can exert anti-tumor, anti-autoimmune, anti-infection, and vaccine adjuvant effects by enhancing immune organ function, boosting immune cell activity, promoting the release of immunoreactive substances, and regulating immune signaling pathways. This review offers a robust theoretical framework and practical guidance for the utilization of tonifying polysaccharides in enhancing immunity and preventing and treating related diseases.

## 2 Pharmacological effects of Tonifying polysaccharides in immunomodulation

### 2.1 Pharmacological effects on immune organs

Immune organs are the core components of the immune system, comprising central immune organs (thymus, bone marrow) and peripheral immune organs (spleen, lymph nodes, and intestine). Central immune organs are the primary sites for the production, proliferation, differentiation, and maturation of immunoreactive cells, and they systematically shape the organization and phenotypic functionality of peripheral immune organs. In contrast, peripheral immune organs are the main sites of immune responses and are responsible for immune surveillance and defense (21).

#### 2.1.1 Spleen and thymus

The spleen and thymus are vital immune organs with distinct functional roles. The spleen, the largest immune organ in the body, acts as a storage and production center for immune cells, such as T and B lymphocytes, and serves as a central hub for triggering innate and adaptive immune responses (22). In contrast, the thymus is primarily responsible for T cell development and maturation, and its core functions are essential for maintaining immune homeostasis (23). The spleen and thymus, with their indices, act as essential indicators and predictors of immune function and prognosis, playing a vital role in immune regulation (24). WANG et al. (25) extracted and purified *Polygonatum sibiricum* polysaccharides (PSP), and isolated four fractions (PSP1–PSP4). The *in vitro* activity was ranked as PSP3 > PSP4 > PSP2 > PSP1. *In vivo* studies found that a high dose (400 mg/kg) of PSP3 significantly increased the splenic index to near-normal levels and regulated the helper T cell 1/helper T cell 2 (Th1/Th2) balance, thereby restoring immune homeostasis. Studies have shown that fructose-rich *Codonopsis pilosula* polysaccharide (LMw-CPP) significantly increased the spleen and thymus indices of cyclophosphamide-immunosuppressed mice in a dose-dependent manner, thereby protecting immune organs (26). SONG et al. (27) found that *Glycyrrhiza* polysaccharide (GP1) could elevate immune organ indices in healthy mice, including indices of the liver and spleen. Additionally, GP1 attenuated the release of pro-inflammatory cytokines (e. g., IL-1β, IL-2, IL-4, IL-10), chemokines (e. g., MIP-1α and MCP-1), and immunoglobulins (e. g., IgG and IgM), demonstrating its potential as an immune dietary supplement. YANG et al. (28) observed that Fuzi polysaccharides (FZPS-1) could significantly increase the spleen and thymus indices in immunosuppressed mice. GAO et al. (8) found that medium and high doses of GLP-1, a low-molecular-weight heteroglycan from *Ganoderma leucocontextum* fruiting bodies, significantly increased thymus indices in immunosuppressed mice, positively influencing the growth and maintenance of immune organs. GAI et al. (29) demonstrated that *Pueraria lobata* polysaccharide (PLP) can raise the ratio of CD4^+^ and CD8^+^ T cells in the spleen and increase serum concentrations of cytokines, such as IL-2, TNF-α, interleukin-4 (IL-4), and interferon-γ (IFN-γ), thereby alleviating immunosuppression.

#### 2.1.2 Bone marrow

Bone marrow serves as the foundation for the growth, development, and maturation of immune cells and plays a central and indispensable role in the construction, maintenance, regulation, and functioning of the immune system (30). It was found that γ-radiation damaged the immune organs of mice and reduced their immune function, while ginseng polysaccharides (GPS-2) increased the number of bone marrow cells and mitigated the damage to bone marrow tissues in radiation-damaged mice (9). XIE et al. (31) demonstrated that polysaccharide-rich extract from *Polygonatum sibiricum* (PREPS) enhanced the number and proportion of bone marrow hematopoietic stem cells (HSCs), long-term HSCs, short-term HSCs, and LSK cells. It also reversed the inhibitory effects of triple-negative breast cancer (TNBC) on bone marrow hematopoietic stem and progenitor cells (HSPCs) and common lymphoid progenitor cells (CLP), and lowered the proportion of immunosuppressive myeloid cells among tumor-infiltrating immune cells, thereby protecting bone marrow hematopoiesis in TNBC mice. ZHANG et al. (32) illustrated that *Astragalus* polysaccharides (APS) attenuated the expression of Bcl-2-associated X protein (Bax), Bcl-2 homologous antagonist/killer (Bak), and cysteinyl aspartate-specific proteinase 3 (Caspase-3), while up-regulating the expression of anti-apoptotic proteins B-cell chronic lymphocytic leukemia/lymphoma 2 (Bcl-2) and BCL2-like 1 (Bcl-xl). These actions regulate mitochondrial pathways and protect bone marrow stromal stem cells (BMSCs) from radiation-induced apoptosis through paracrine mechanisms. Bone marrow immunity is crucial to the development of osteoporosis (OP) by directly influencing osteoblast activity, maintaining microenvironmental homeostasis, and mediating inflammatory responses (33). SONG et al. (34) found that *Achyranthes bidentata* polysaccharide (ABP) effectively blocked receptor activator of nuclear factor-κB ligand (RANKL)-induced osteoclastogenesis, resulting in fewer osteoclasts and diminished bone resorption activity. Additionally, ABP down-regulated the expression of nuclear factor of activated T-cells, cytoplasmic, calcineurin 1 (NFATc1), c-Fos, and bone resorption-related proteins, and inhibited the phosphorylation of JNK1/2, ERK1/2, and p38. These effects blocked the NFATc1 and mitogen-activated protein kinase (MAPK) signaling pathways, thereby preventing osteoporosis. Investigations have revealed that *Astragalus* polysaccharide (APS) (35) stimulates cell proliferation by augmenting cyclin D1 and promotes osteogenesis by up-regulating osteogenic markers, including alkaline phosphatase (ALP), osteocalcin (OCN), and RUNX family transcription factor 2 (RUNX2), as well as inducing the formation of calcium nodules. Meanwhile, APS inhibited the expression of miR-760 and increased the expression of ankyrin repeat and FYVE domain-containing protein 1 (ANKFY1), ultimately promoting osteogenic maturation and growth of human BMSCs and providing a new therapeutic strategy for osteoporosis.

#### 2.1.3 Intestine

The primary site of intestinal immunity is the mucosa-associated lymphoid tissue (MALT), which includes structures like Peyer’s patches, the appendix, and mesenteric lymph nodes. YANG et al. (36) demonstrated that *Coptis chinensis* polysaccharides (CCPs) can be endocytosed by microfold cells (M-cells) on the surface of Peyer’s patches, thereby activating lymphocytes and regulating the Th1/Th2 balance. Additionally, CCPs activate the TLR4/NF-κB signaling pathway and regulate the release of immune mediators, including IFN-γ and IL-4, thereby dynamically regulating the small intestinal microenvironment. Intestinal flora may regulate the maturation of mucosal immunity, while pathogenic microorganisms can trigger immune dysfunction (37). YANG et al. (38) demonstrated that *Hericium erinaceus* polysaccharides (HEPs) can enhance the richness and diversity of the intestinal flora by modulating the balance of intestinal probiotics. For example, HEPs significantly enhanced the relative abundance of Lachnospiraceae and Akkermansiaceae, while diminishing the relative abundance of Rikenellaceae and Bacteroidaceae, thereby modulating the intestinal immune response. DONG et al. (39) demonstrated that *Lactarius deliciosus* polysaccharides (LDPs) can improve intestinal flora diversity in tumor-bearing mice by augmenting the richness of Lactobacillaceae and Lactobacillus, while lowering the abundance of Prevotellaceae and Alloprevotella. Additionally, a high dose of LDP (100 mg/kg) can promote adenine-mediated zeatin biosynthesis by regulating intestinal metabolic pathways and modulating receptor-related metabolic activities, such as estrogen, thereby exerting a tumor immunomodulatory effect. ZHANG et al. (40) found that both *Cyclocarya paliurus* polysaccharides (CPP0.1) and acetylated *Cyclocarya paliurus* polysaccharides (Ac-CPP0.1) modulated immune function in immunosuppressed mice by enhancing cytokine release, including IL-4, IL-17, transforming growth factor-β3 (TGF-β3), and TNF-α, as well as the expression of related gene mRNAs in serum and small intestine. The influence of Ac-CPP0.1 was more pronounced. Meanwhile, both polysaccharides likewise elevated the levels of short-chain fatty acids (SCFAs) in the cecal contents of mice, with Ac-CPP0.1 having a more pronounced influence on the enhancement of individual fatty acids and total fatty acids. It was demonstrated that purified hyssop *Achyranthes bidentata* polysaccharide (ABPW1) could alleviate kidney injury and inflammation in diabetic mice by modulating gut microbial composition, increasing plasma and fecal levels of propionic acid and isobutyric acid, and enhancing SCFA production (41).

### 2.2 Pharmacological effects on immune cells

Immune cells originate from immune organs and undergo differentiation, maturation, and immune response processes to identify and eliminate pathogens (e.g. bacteria and viruses) and abnormal cells (e.g. cancer cells). The major immune cell types include T and B lymphocytes, macrophages, natural killer cells, and dendritic cells (21). Tonifying polysaccharides enhance immune function by modulating immune cell activity, thereby benefiting immune-related diseases.

#### 2.2.1 T and B lymphocytes

Lymphocytes, which constitute the principal effector cells within the immune system, are essential for specific immune responses. They are capable of differentiating and proliferating upon antigen stimulation, a process critical for their function. Normal lymphocyte percentages range from 20%-40%, and their counts should be between 0.8 to 4 x 10^9 per liter of blood. Their proliferative capacity is an important biological indicator for assessing the strength of the immune response (42). During their differentiation and maturation, T lymphocytes can differentiate into various subpopulations with distinct functions, including helper T cells (Th cells, characterized by the CD4+ marker), cytotoxic T cells (Tc cells or CTL cells, identified by the CD8+ marker), and regulatory T cells (Treg cells, which can also be marked by CD4+ in some cases). Among them, Th cells can be further categorized into functional subsets, including Th1, Th2, Th17, Tfh, and Treg. Th1 cells mediate cellular immune responses through the secretion of IL-2 and IFN-γ, whereas Th2 cells enhance humoral immunity via the secretion of IL-13, IL-4, and IL-5. Both promote the activation of cytotoxic T cells and B cells (43). Maintaining normal immune function relies on the balance of T cell subset ratios. For example, an elevated CD4^+^/CD8^+^ T-cell and ratio indicate an immune-activated state, whereas a decreased ratio indicates an immune-suppressed state. Feng et al. (25) discovered that the root extract of Panax ginseng C. A. Meyer contains three polysaccharide fractions: GPS-1a, GPS-1b, and GPS-2. Among them, GPS-2 exhibited significant immunomodulatory effects, markedly boosting splenic lymphocyte proliferation and augmenting the CD4^+^/CD8^+^ T-cell ratio, outperforming GPS-1a and GPS-1b. In a study of GPP, a complex of *Ganoderma lucidum* polysaccharides (GLP) and *Polyporus umbellatus* polysaccharides (PUP) (44) formulated at a 3:1 ratio, low (36 mg/g) and medium (120 mg/g) doses of GPP significantly enhanced lymphocyte proliferation, whereas high (360 mg/g) doses did not promote lymphocyte proliferation. These findings suggest that GPP promotes lymphocyte proliferation and, consequently, cellular immunity only within a specific concentration range. YANG et al. (45) revealed that *Alpiniae oxyphyllae* fructus polysaccharides contain three fractions, AOFP-1, AOFP-2, and AOFP-3, all of which regulate immune functions by stimulating lymphocyte proliferation. Among these, AOFP-1 exhibited a particularly significant immunomodulatory effect, surpassing AOFP-2 and AOFP-3. AOFP-1 significantly elevated the levels of IL-2 and interferon-γ, inducing a Th1-type immune response, and also significantly promoted IL-4 and IL-6 secretion, activating a Th2-type immune response. LEI et al. (46) demonstrated that *Achyranthes bidentata* polysaccharides (ABPs) could exert immunomodulatory effects by significantly promoting the secretion of IL-2, IFN-γ, TNF-α, and IL-6, thereby enhancing the Th1 immune response and inhibiting Th2 differentiation.

B lymphocytes originate from bone marrow-resident hematopoietic stem cells and modulate adaptive immune responses through antibody production and antigen presentation (47). Immunoglobulins (IgM, IgD, IgG, IgA, and IgE) are surface markers of B lymphocytes and are primarily responsible for antigen recognition. LIANG et al. (48) discovered that both fermented *Lentinus edodes* polysaccharide (F-LEP-2a) and non-fermented *Lentinus edodes* polysaccharide (NF-LEP-2a) exhibited significant immunomodulatory effects, elevating serum levels of IgG and IgM, with F-LEP-2a showing a more pronounced effect at a high dose (400 mg/kg). JI et al. (49) showed that *Salvia miltiorrhiza* polysaccharides (SMEPs) dose-dependently increased the proportion of CD19^+^ B cells in immunosuppressed mice and significantly elevated the percentage of CD3^+^ T cells, thereby enhancing both cellular and humoral immune reactions. GAO et al. (8) focused on GLP-1, a low-molecular-weight heteropolysaccharide from Ganoderma leucocontextum fruiting bodies, and found that GLP-1 increased serum IgM and IgG levels in a dose-dependent manner in immunosuppressed mice, thereby boosting humoral immune function. SU et al. (50) investigated the relationship between the number of times *Polygonatum* was steamed and its immunomodulatory effects. They found that both polysaccharides obtained from *Polygonatum* after six times of steaming (SYWPP) and nine times of steaming (NYWPP) significantly enhanced immune responses, including up-regulation of interleukin-2 (IL-2), IFN-γ, IgM, and IgA levels, and augmented the CD4^+^/CD8^+^ T cell ratio. SYWPP treatment was particularly effective in immunomodulation, confirming the optimal immunomodulatory effect of polysaccharides obtained from six times of steaming *Polygonatum*.

#### 2.2.2 Macrophages

Macrophages, as essential multifunctional cells within the immune system, are extensively dispersed across various tissues and organs throughout the body. They exhibit high plasticity and can polarize into different phenotypes elicited by microenvironmental cues, primarily categorized as M1-type and M2-type macrophages. M1-type macrophages mainly secrete pro-inflammatory factors such as IL-6, IL-12, IL-1β, TNF-α, and iNOS. They also display surface markers, including CD86, CD80, and MHC class II molecules. These markers mediate antibacterial, anti-tumor, and pro-inflammatory functions. Conversely, M2-type macrophages release inflammation-suppressing cytokines (e. g., EGF, TGF-β, IL-10) and express surface markers such as CD163 and CD206, which are involved in anti-inflammatory effects, tissue repair, tumor promotion, and immune regulation (51). Experimental results have indicated that *Ganoderma leucocontextum* polysaccharide (GLP-3) (52) elevates nitric oxide (NO) content and the levels of several cytokines, such as TNF-α, IL-6, IL-1α, IL-1β, and IL-10, in RAW264.7 macrophages, thereby regulating the balance of M1/M2 macrophages. Meanwhile, GLP-3 stimulates the secretion of M1-type chemokine CXCL5 and M2-type chemokines (e. g., MIP-2, MCP-1), which promote Th1- and Th2-type immune responses, thereby achieving immunomodulatory effects through multiple pathways. LI et al. (53) extracted and purified three polysaccharide fractions from *Codonopsis pilosula*: CPPS-I, CPPS-II, and CPPS-III. All fractions demonstrated inhibitory effects on tumor growth, with CPPS-II demonstrating the most significant activity. CPPS-II significantly promoted NO secretion and CD86 expression and induced macrophage polarization from the M0 to the M1 phenotype, thereby enhancing tumor cell apoptosis. Additionally, when combined with *Adriamycin*, CPPS-II exhibited a superior anti-tumor effect compared to *Adriamycin* alone. Following the combination, the M1/M2 ratio increased, CD206 expression (an M2 marker) was down-regulated, and iNOS (inducible nitric oxide synthase, an M1 marker) expression was up-regulated, thereby exerting anti-tumor immune effects through regulation of macrophage polarization. ZHOU et al. (54) found that *Polygonatum sibiricum* polysaccharides (PSPs) down-regulated the M1 marker iNOS while up-regulating the M2 marker CD206, thereby inhibiting M1 polarization and promoting an anti-inflammatory M2 phenotype. This action reduced lung inflammation and modulated macrophage polarization.

Additionally, macrophages enhance immune function by releasing cytokines, chemokines, and inflammatory factors (55). JI et al. (56) used ultrasound-assisted extraction to obtain *Codonopsis pilosula* polysaccharides (CPPs) and found that CPPs dose-dependently stimulated the secretion of NO, IL-6, and TNF-α from macrophages without cytotoxicity, suggesting that CPPs are a natural immune-stimulating supplement. *Angelica dahurica* polysaccharide (ADP 80-2) (57), composed of arabinose and glucose, enhanced phagocytosis and induced the secretion of IL-6, IL-1β, and TNF-α by macrophages, as verified by RAW264.7 macrophage assays. This indicates significant immunomodulatory activity and potential as an immunomodulator. TIAN et al. (58) showed that pectinase hydrolysis of polysaccharides from *Tetrastigma hemsleyanum* Diels et Gilg resulted in the isolation of STHP4–1 and STHP4-2. Among them, STHP4–1 was the most effective in inducing NO production and increasing cytokine concentrations (e. g., IL-6, TNF-α, and IL-1β) in macrophages, confirming its potent immunomodulatory activity.

#### 2.2.3 Natural killer cells

Natural Killer Cells (NKCs) originate from the bone marrow and serve as key effector cells of innate immunity, primarily guarding against infections and tumors. First, the signaling balance between activating receptors (e. g., recognizing MICA/B) and inhibitory receptors (e. g., recognizing MHC-I) determines whether the cell is activated. Secondly, once activated, NKCs directly eliminate target cells and modulate the immune response by secreting perforin, granzymes, and cytokines such as IFN-γ and TNF-α, thereby performing anti-tumor and antiviral functions (59). YU et al. (26) found that fructose-rich *Codonopsis pilosula* polysaccharide (LMw-CPP) significantly enhanced the cytotoxic efficacy of Natural Killer Cells (NKCs) in a dose-dependent manner, thereby exerting an immunomodulatory effect. LI et al. (44) investigated GPP, a complex of *Ganoderma lucidum* polysaccharides (GLP) and *Polyporus umbellatus* polysaccharides (PUP) formulated at a 3:1 ratio, and found that GPP substantially augmented the phagocytosis rate and phagocytic index of DSS (dextran sodium sulfate)-induced macrophages in mice. Additionally, GPP significantly augmented the cytotoxicity of NK cells against YAC-1 tumor cells in DSS-induced mice at high doses, thereby boosting NK cell activity and enhancing the innate immune response in colitic mice. JI et al. (49) discovered that *Salvia miltiorrhiza* polysaccharides (SMEPs) dose-dependently boosted the cytotoxicity of Natural Killer (NK) cells in cyclophosphamide-treated mice, substantially up-regulating the transcription of TNF-α, IFN-γ, IL-2, and IL-4, thereby enhancing immune defenses. GE et al. (60) confirmed that *Angelica gigas* polysaccharide (F2 polysaccharide) activated NK-92 cells, augmented their cytotoxic activity toward HCT-116 cells, and promoted the transcription of TNF-α, IFN-γ, NKp44, and Granzyme-B, thereby exerting anti-tumor immune activity through the MAPK and NF-κB signaling cascades. YANG et al. (61) showed that *Cnidium officinale* polysaccharide enhanced the cytotoxicity of NK-92 cells against HCT-116 cells and up-regulated the expression of IFN-α, TNF-α, NKp44, and Granzyme-B, thereby activating the immunomodulatory effects of NK-92 cells.

#### 2.2.4 Dendritic cells

**Figure 1:**
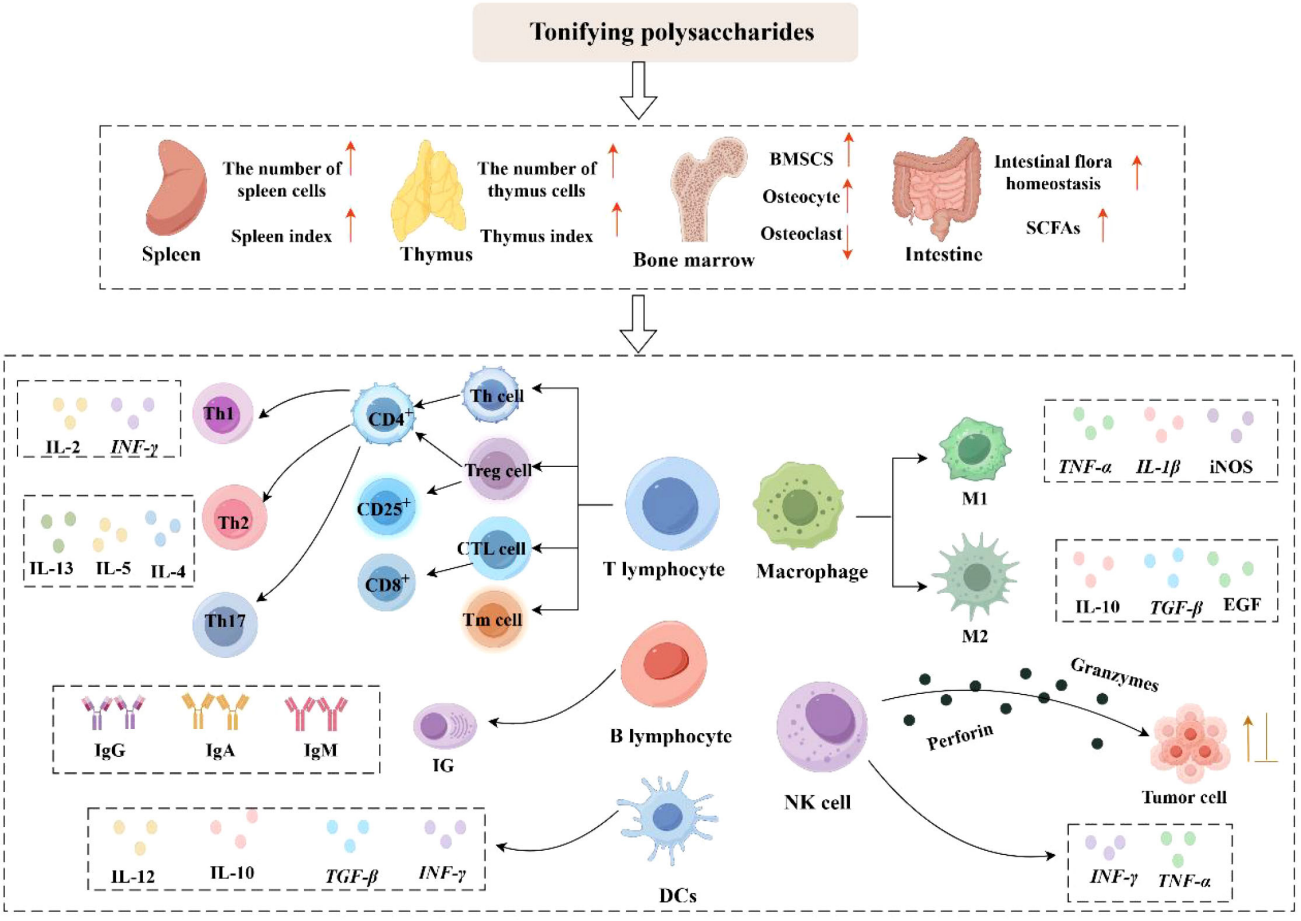
Immunomodulatory effects of Tonifying polysaccharides (activation: ↑; inhibition: ⊥) (By Figdraw).

Dendritic cells (DCs) originate from bone marrow hematopoietic stem cells and are the most potent specialized antigen-presenting cells in the body. Based on their phenotypic characteristics and functional differences, dendritic cells can be categorized into three distinct subsets: plasmacytoid dendritic cells (pDCs), conventional dendritic cells (cDCs), and monocyte-derived dendritic cells (moDCs). Conventional dendritic cells are further subdivided into two subpopulations: cDC1 and cDC2. Dendritic cells release diverse cytokines and chemokines (e. g., IL-12, IL-10, TGF-β, IFN-γ) and display elevated levels of co-stimulatory molecules such as CD80 and CD86 on their surface. These molecules effectively activate T lymphocytes through antigen presentation, thereby modulating the onset and trajectory of adaptive immune reactions (62). Studies have shown that *Chrysanthemum zawadskii* Herbich var. *latilobum* polysaccharide (CP) (63) can induce the expression of surface molecules (such as CD80, CD86, and MHC) on dendritic cells, thereby activating the MAPK and NF-κB signaling pathways to induce the maturation of dendritic cells. Additionally, CP-treated dendritic cells significantly promoted the proliferation of T cells and elevated the secretion of IFN-γ and IL-2, thereby contributing to the Th1-type immune response and enhancing the overall immune response. WANG et al. (64) demonstrated that Isatis root polysaccharide (IRPS) exerts immune-enhancing and antiviral functions by altering IL-12 and IL-6 levels, promoting dendritic cells’ maturation and Th1-type immune responses, and enhancing antigen presentation while alleviating immunosuppression by pseudorabies virus (PRV). HE et al. (65) found that microparticles composed of cationic-modified calcium carbonate (CaCO_3_) and encapsulated with *Polygonatum sibiricum* polysaccharide (PEI-PSP-CaCO_3_) significantly increased the expression of CD80, CD86, and CD40 on the dendritic cells’surface, promoted antigen transport to draining lymph nodes, and enhanced immunomodulatory activity. *Lycium barbarum* polysaccharides liposomes (LBPL) (66) upregulated the expression of co-stimulatory molecules, promoted cytokine production (e. g., IL-12, TNF-α), and enhanced antigen internalization. Meanwhile, LBPL stimulates the activation and maturation of immature dendritic cells (DCs) through the TLR4-MyD88-NF-κB pathway. QIAO et al. (67) found that *Selenizing Chinese Angelica* polysaccharide (sCAP) exhibited a stronger immune-activating effect than *Chinese Angelica* polysaccharide (CAP). sCAP promoted the proliferation and maturation of immature bone marrow-derived dendritic cells (BMDCs), as demonstrated by upregulation of CD40 and MHC II, enhanced their ability to activate lymphocytes, and regulated cytokine levels, including elevated IL-6 and TNF-α, and decreased IL-1β and IL-10. The studies demonstrate that modified polysaccharides retain immunomodulatory activity and can induce phenotypic changes in dendritic cells, thereby driving their maturation. **Figure 1** illustrates the immune-regulating activities of Tonifying polysaccharides.

## 3 Mechanisms of action of Tonifying polysaccharides in immunomodulation

### 3.1 NF-κB signaling pathway

The NF-κB family includes core proteins p50, p65, RelB, c-Rel, and p52. NF-κB remains in the cytoplasm during the resting phase. Upon external stimulation, the signaling pathway is activated, leading to the activation of the IκB kinase (IKK) complex. Activation of IKK leads to the phosphorylation of IκB, which in turn results in IκB degradation and the subsequent release of NF-κB dimers. These dimers translocate to the nucleus, where they modulate inflammation, cell growth, and immune responses (68). SONG et al. (69) observed that treatment with alkali-extracted *Bupleurum chinense* polysaccharide (BCAP-1) in homozygous mice significantly inhibited the growth of sarcoma 180, promoted TNF-α, NO, and iNOS secretion, and enhanced macrophage phagocytic activity. Meanwhile, BCAP-1 activated NF-κB signaling via p65 phosphorylation and reduced IκB expression in macrophages, thereby exerting its anti-tumor immune regulatory mechanism. QIN et al. (70) identified that sHEP2 in *Selenizing Hericium erinaceus* polysaccharides (sHEPs) induced dendritic cell (DC) maturation and exerted immunomodulatory effects by inducing IκBα degradation, promoting the nuclear translocation of p65 and p50, and enhancing NF-κB signaling pathway activity. YANG et al. (61) demonstrated that *Cnidium officinale* polysaccharide significantly promoted NO secretion and up-regulated the expression of TNF-α, IL-1β, and IL-6 in RAW264.7 macrophages, while also activating p65 phosphorylation and exerting immunomodulatory effects via the NF-κB signaling cascade.

The NF-κB signaling pathway is regulated by various receptors, including Toll-like receptors (TLRs), the tumor necrosis factor receptor (TNFR) family, and the interleukin-1 receptor (IL-1R). TLRs are vital for innate immunity and activate downstream NF-κB and mitogen-activated protein kinases (MAPKs) via myeloid differentiation factor 88 (MyD88), prompting cells to synthesize and release large amounts of inflammatory cytokines (e. g., TNF-α, IL-1β, IL-6), chemokines, and interferons, thereby regulating immune signaling (71). WU et al. (72) uncovered that *Tetrastigma hemsleyanum* polysaccharide (THP) triggers specific signaling cascade reactions through its action on TLR4, resulting in strong p65 phosphorylation and weak IκBα phosphorylation, and significantly increases the relative values of p-p65/p65 and p-IκBα/IκBα. Meanwhile, THP promotes p65 nuclear translocation, thereby activating the NF-κB pathway. *Lycium barbarum* polysaccharides liposomes (LBPL) (66) exert immunomodulatory effects by engaging the TLR4 receptor, initiating the MyD88-dependent pathway, inducing TRAF6 phosphorylation, and thereby triggering the NF-κB pathway, thereby which facilitates dendritic cell (DC) maturation and Th1-type immune polarization. ZHANG et al. (73) revealed that *Catharanthus roseus* polysaccharides (CRPS-2) activate the NF-κB pathway by dose-dependently elevating TLR2 and TLR4 expression, which is one of the important mechanisms underlying its immunomodulatory effects.

### 3.2 MAPK signaling pathway

The mitogen-activated protein kinase (MAPK) signaling pathway is a three-tiered kinase cascade mechanism, primarily composed of MAPK kinase kinase (MAP3K), MAPK kinase (MAP2K), and MAPK. This pathway impacts inflammation, cell differentiation, proliferation, apoptosis, immune regulation, and stress response (74). The MAPK signaling pathway consists of three core pathways: the c-Jun N-terminal kinase (JNK) pathway, the p38 MAPK pathway, and the extracellular signal-regulated kinase (ERK) pathway (75). Different pathways regulate distinct cellular processes: the extracellular signal-regulated kinase (ERK) pathway predominantly responds to growth-promoting cytokines, thereby stimulating cell proliferation and differentiation; whereas the JNK and p38 pathways are associated with cellular stress responses and immuno-inflammatory processes. The three pathways in the MAPK pathway—ERK, JNK, and p38—are both independent and interact with each other, forming a complex regulatory network. GUO et al. (15) verified that *Ganoderma lucidum* polysaccharide (GLP) inhibits the phosphorylation of JNK and ERK triggered by lipopolysaccharides in both normal intestinal epithelial cells (NCM460) and colorectal cancer cells (HT-29), attenuates the expression levels of inflammatory mediators (e. g., iNOS, COX-2, IL-1β, TNF-α), and thereby inhibits the MAPK pathway, exerting inflammation-suppressing and anti-tumor effects. QIN et al. (70) found that *selenized Hericium erinaceus* polysaccharides (sHEPs) significantly enhanced the phosphorylation of ERK, JNK, and p38, and promoted the nuclear translocation of p-c-Jun, p-CREB, and c-Fos, thereby promoting dendritic cell (DC) proliferation and maturation and exerting immunomodulatory effects. *Tetrastigma hemsleyanum* polysaccharide (THP) treatment (72) significantly elevated the phosphorylation of ERK, JNK, and p38 MAPK in macrophages, indicating its potential to modulate immune responses via these MAPK pathways. ZHANG et al. (73) revealed that *Catharanthus roseus* polysaccharides (CRPS-2) promote the phosphorylation of ERK, JNK, and p38 proteins in a dose-dependent manner, thereby activating the MAPK pathway, which is one of the key mechanisms underlying its immunomodulatory effects.

### 3.3 PI3K/Akt signaling pathway

The PI3K/Akt signaling pathway serves as a crucial modulator of cell growth, proliferation, differentiation, and metabolic processes (76). The canonical PI3K/Akt pathway is activated by the activation of phosphatidylinositol-3 kinase (PI3K) upon exposure to extracellular stimuli. Upon activation, PI3K catalyzes the conversion of phosphatidylinositol-4,5-bisphosphate (PIP2) to phosphatidylinositol-3,4,5-trisphosphate (PIP3). PIP3 functions as an intracellular signal transducer, recruiting and activating protein kinase B (Akt) to the plasma membrane, thereby initiating a cascade of downstream reactions and regulating immune function. HU et al. (77) investigated the immunomodulatory activities of longan polysaccharide (CP1) and its fermentation products, including longan wine (CP2), vinegar (CP3), and CP4. The findings indicated that the CP3 vinegar fermentation product augmented the phosphorylation of PI3K p85, IκBα, and Akt in macrophages, while also stimulating cytokine release, including NO, TNF-α, and IL-6, thereby activating the immune response. Additionally, the immunomodulatory effects of CP3 are strongly associated with its low molecular weight and increased mannose and arabinose content. ZHANG et al. (73) revealed that *Catharanthus roseus* polysaccharides (CRPS-2) activate the Akt/mTOR (mammalian target of rapamycin) pathway through enhanced expression of phosphorylated Akt and mTOR, which is one of the key mechanisms underlying its immunomodulatory activity. QI et al. (78) observed that low-molecular-weight ginseng polysaccharides (LGP) alleviated spontaneous liver injury through suppression of the PI3K/Akt pathway. LGP inhibited the phosphorylation of PI3K, Akt, and mTOR proteins and reduced the mRNA expression of inflammatory mediators (e. g., IL-1β, IL-6, IL-18, TNF-α), thereby attenuating inflammation. Additionally, LGP diminished Bax expression, augmented Bcl-2 levels, and subsequently reduced the Bax/Bcl-2 ratio, thereby reducing hepatocyte apoptosis. WU et al. (79) showed that *azuki bean (Vigna angularis*) polysaccharide (ABP) significantly up-regulated the gene and protein expression of insulin receptor (INSR), insulin receptor substrate-1 (IRS-1), phosphatidylinositol-3 kinase (PI3K), protein kinase B (Akt), and glucose transporter 2 (GLUT-2) in the liver of diabetic rats. This mechanism involves activation of the insulin/PI3K/Akt pathway, thereby promoting glucose metabolism and maintaining immune homeostasis.

**Figure 2:**
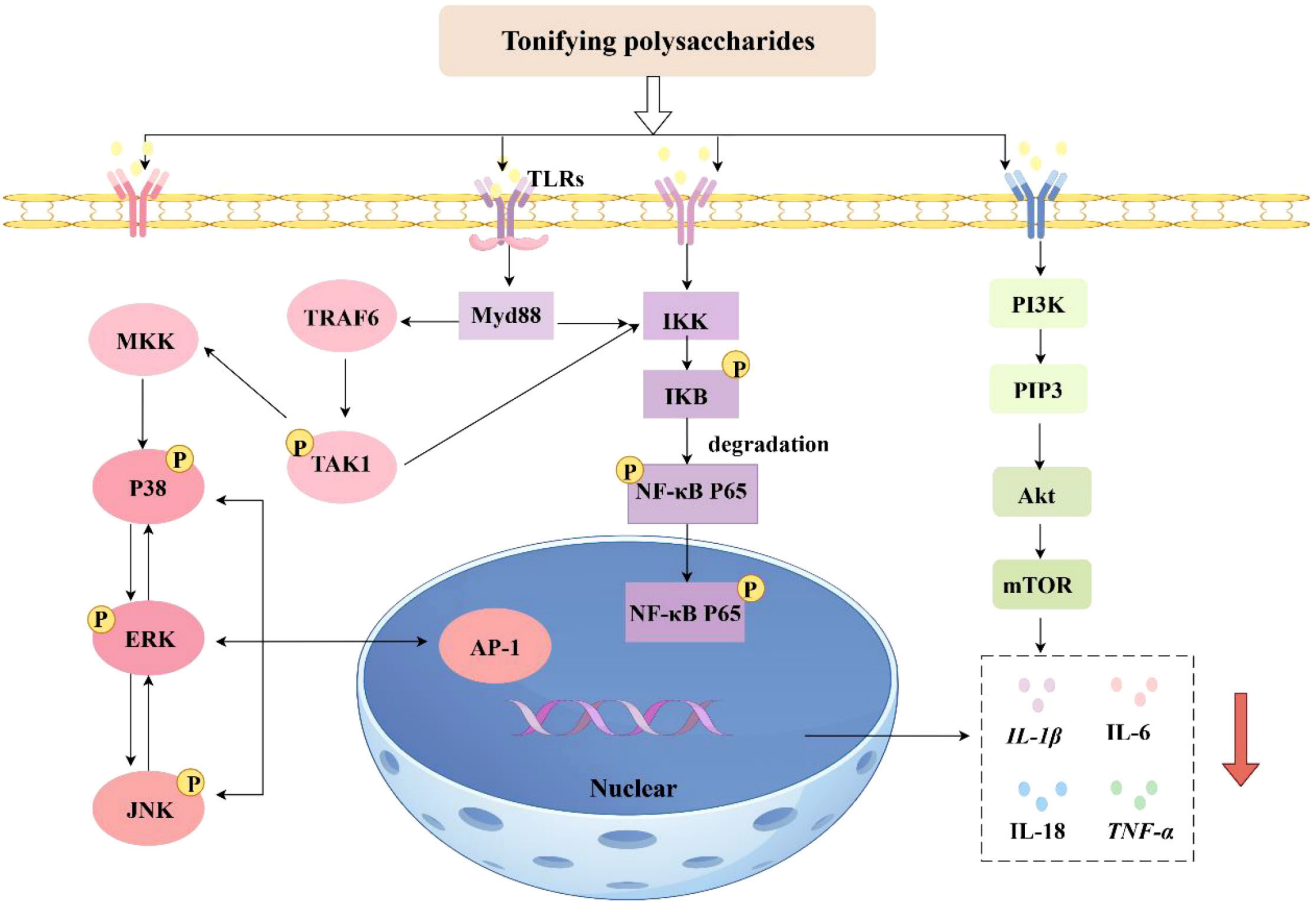
Mechanisms of immunomodulatory effects of Tonifying polysaccharides (inhibition: ↓) (By Figdraw).

In conclusion, existing studies have demonstrated that both the NF-κB signaling pathway and the mitogen-activated protein kinase (MAPK) signaling pathway are involved in the pathophysiological processes of inflammatory diseases and tumors (80). However, aberrant activation of the PI3K/Akt signaling pathway is closely associated with tumor development and the pathogenesis of metabolic diseases. The NF-κB signaling cascade predominantly governs the synthesis of pro-inflammatory cytokines at the transcriptional level. Among the MAPK pathways, the p38 MAPK and JNK signaling pathways are strongly correlated with neuroinflammation, apoptosis, and glial scarring. The phosphatidylinositol 3-kinase/protein kinase B (PI3K/Akt) pathway is implicated in neuroprotection, cell survival, and axonal regeneration (74). The immunomodulatory mechanisms of Tonifying polysaccharides are depicted in **Figure 2**.

## 4 Therapeutic application of Tonifying polysaccharides in immune-related diseases

### 4.1 Anti-tumor

The tumor microenvironment (TME) significantly influences tumor development and progression. It enables tumor cells to evade immune surveillance and clearance through multiple mechanisms, thereby constructing an immunosuppressive microenvironment and promoting tumor cell proliferation, invasion, and metastasis. Investigations have revealed that tonifying polysaccharides possess the capacity to modulate the tumor microenvironment through multiple pathways, including stimulating the polarization of tumor-associated macrophages to the M1 phenotype; enhancing the maturation of dendritic cells and their antigen-presenting ability; enhancing the cytotoxic and proliferative activities of natural killer cells; and modulating T-lymphocyte activation, proliferation, and cytokine secretion. Through these mechanisms, Tonifying polysaccharides can remodel the tumor microenvironment, reverse its immunosuppressive state, and stimulate the body’s anti-tumor immune response, thereby exerting anti-tumor immunomodulatory effects (62, 80).

Macrophages polarized to the M1 phenotype suppress tumor growth, while those polarized to the M2 phenotype facilitate tumor progression (81). Therefore, shifting macrophage polarization to the M1 phenotype has emerged as a potential approach in cancer immunotherapy. Research has demonstrated that *Astragalus* polysaccharide (PG2) (82) markedly elevated the M1/M2 polarization ratio and suppressed the levels of pro-tumor cytokines IL-6 and IL-10 in non-small cell lung cancer cell lines H1299 and H441. Additionally, PG2 promotes dendritic cell (DC) maturation, thereby enhancing T-cell activity and mediating antitumor immune regulation. Clinical studies have shown that *Astragalus* polysaccharide injection (PG2) significantly reduces the neutrophil-lymphocyte ratio (NLR) in advanced lung cancer patients receiving immune checkpoint inhibitor therapy, especially in those with a high baseline NLR. Moreover, PG2 modulates macrophage polarization (M1/M2) and dendritic cell maturation, thus enhancing antitumor immunity (83). LIU et al. (84) confirmed that the combination of *Ganoderma lucidum* crude polysaccharides (GLCP) and *Codonopsis pilosula* crude polysaccharides (CPCP) followed by dacarbazine significantly reduced CD68^+^ macrophage numbers in murine melanoma tissue and inhibited tumor growth. Additionally, particular concentrations of digested *Codonopsis pilosula* polysaccharide (dCPP) enhanced the expansion of M1-type macrophages, inhibited the proliferation of M2-type macrophages, and induced the polarization of M2-type macrophages to the M1 phenotype by up-regulating the transcriptional levels of IL-1, IL-6, iNOS, and TNF-α, thereby inhibiting melanoma progression. Tonifying polysaccharides can regulate neutrophil polarization, inhibit the expression of pro-tumor phenotypes (N2 type), and promote the expression of anti-tumor phenotypes (N1 type), thereby exerting a substantial influence on anti-tumor immune regulation. XU et al. (85) demonstrated that *Lentinan* (LNT), either alone or in combination with Delta-like 1 (DLL1), inhibited the growth of both breast and lung cancer, with the combined treatment yielding superior results. Additionally, LNT enhances the antitumor effects of DLL1 through two immune mechanisms: it promotes the infiltration and activation of CD8^+^ T cells, thereby activating adaptive immunity, and induces neutrophils to polarize toward the N1 type, secreting cytokines such as IFN-γ to synergize with tumor suppression and remodel innate immunity. ZHAO et al. (86) demonstrated that the co-administration of *Lentinan* and cisplatin thoracic injection significantly enhances the clinical efficacy in patients with non-small cell lung cancer (NSCLC) and improves their quality of life. *Lentinan* has been demonstrated to stimulate immune cells such as T cells and NK cells, promote the secretion of cytokines including IL-6 and TNF-α, and inhibit the expression of VEGF. The combined mechanisms of immune regulation and anti-angiogenesis have been shown to yield significant clinical benefits in cancer treatment.

The maturation and migration of dendritic cells (DCs) activate T cells and significantly enhance the anti-tumor immune response (87). ZHANG et al. (88) found that *Lonicera japonica* thunb. polysaccharides (LJP)-exosomes significantly promoted polysaccharide delivery. LJP-exosomes not only enhanced the migration and maturation of DCs in 4T1 hormonal mice but also stimulated pro-inflammatory cytokine secretion and upregulated MHC-II expression in lymph nodes. Additionally, LJP-exosomes significantly inhibited 4T1 tumor growth, enhanced the infiltration of DCs, CD4^+^ T cells, and CD8^+^ T cells, while reducing the percentage of regulatory T cells (Tregs) within the tumor, thereby improving the immunosuppressive tumor microenvironment and enhancing LJP transport and immune system activation. LAN et al. (89) investigated the *Fructus corni* acidic polysaccharide (FCP-3) complex and successfully prepared polysaccharide-selenium nanoparticle composites (FCP-SeNPs). The results showed that both FCP-3 and FCP-SeNPs effectively promoted the secretion of NO, TNF-α, and IL-12 from macrophages and increased the CD4^+^/CD8^+^ T cell ratio. Additionally, FCP-3 and FCP-SeNPs inhibited tumor growth and proliferation and promoted apoptosis of hepatocellular carcinoma cells, with FCP-SeNPs being more effective than FCP-3 alone, thereby playing a significant role in hepatocellular carcinoma immunomodulation. DONG et al. (39) demonstrated that high-dose treatment with *Lactarius deliciosus* polysaccharides (LDPs) significantly inhibited tumor growth (tumor suppression rate, 51.61%) and elevated the percentages of CD3^+^, CD4^+^, and CD8^+^ T cells within the peripheral blood of mice bearing tumors. The findings suggest that the antitumor immune function of LDPs is related to T-cell activation. QIAN et al. (90) found that purified ginger polysaccharide (UGP1) markedly increased the expression levels of TNF-α, IL-2, IL-6, and other pro-inflammatory mediators, decreased levels of TGF-β and bFGF (basic fibroblast growth factor), and other pro-tumorigenic factors, and induced apoptosis by regulating the p53, caspase-3, and Bax/Bcl-2 ratio, thereby effectively suppressing the proliferation of human colon cancer cells.

### 4.2 Anti-autoimmune diseases

Autoimmune diseases are conditions in which the immune system mounts a pathologic immune response against self-antigens, leading to tissue injury and impaired function. The pathogenesis of autoimmune diseases involves multiple factors, including abnormal function of immune organs, dysregulated activation of immune cells, and cytokine network imbalance. Studies have shown that Th17 cells promote inflammatory responses and enhance resistance to certain pathogens but are also closely associated with the development of diverse autoimmune disorders, such as psoriasis and inflammatory bowel disease. Treg cells regulate the activation and expansion of effector T cells and inhibit excessive immune responses, thereby preventing autoimmune diseases (91). Polysaccharide from *Atractylodes macrocephala* Koidz (PAMK) (92) significantly alleviated weight loss, disease activity index (DAI) scores, and colon shortening in mice, and down-regulated the expression of pro-inflammatory cytokines, thereby inhibiting inflammation and alleviating pathological damage to the colon. Additionally, PAMK reduced the proportion of Th17 cells and their manifestation of related cytokines in mice with colitis, increased the expression of Treg-related factors, and modulated the Th17/Treg equilibrium, thus exerting a therapeutic effect on ulcerative colitis (UC) in mice. HAO et al. (93) investigated the diverse pathways through which ginger polysaccharide (GP) mitigates ulcerative colitis (UC) symptoms in mice. GP protects the intestinal barrier integrity by repairing the DSS-induced decrease in the expression of tight junction proteins, zona occludens 1 (ZO-1) and occludin-1, and by decreasing apoptosis of colonic epithelial cells. Additionally, GP significantly down-regulated the expression of inflammatory cytokines (e. g., TNF-α, IL-6, IL-1β, IL-17A, IFN-γ) in colonic tissues, thereby suppressing the abnormal inflammatory reaction of UC. These results suggest that Tonifying polysaccharides can alleviate ulcerative colitis (UC) in mice, with mechanisms including immune cell regulation, inflammatory cytokine modulation, increased intestinal flora diversity, and maintenance of the intestinal wall’s immune barrier function (94). Psoriasis is a chronic inflammatory autoimmune skin disease characterized by an imbalance between innate and adaptive immunity, which interact to form an inflammatory cascade response, leading to excessive proliferation of skin keratinocytes, abnormal differentiation, and epidermal inflammation (95). *Punica granatum* peel polysaccharides (PPPs) (96) reduce the psoriasis area and severity index (PASI) and transepidermal water loss (TEWL) by significantly improving psoriasis-like skin lesions, downregulating the expression of Ki67 and CD3, inhibiting the levels of pro-inflammatory cytokines (e. g., TNF-α, IL-6, IL-1β, IL-17, IL-23) in serum, and upregulating the mRNA and protein expression of aquaporin-3 (AQP3) and filaggrin (FLG) in mouse skin. These actions regulate immune balance and skin barrier function, thereby contributing to psoriasis treatment. Allergic asthma is a type 2 immune-mediated chronic inflammatory condition characterized by airway inflammation, mucus hypersecretion, and epithelial barrier dysfunction (97). *Lentinan* (LTN) (98) markedly diminished the quantity of eosinophils and neutrophils in bronchoalveolar lavage fluid (BALF) and decreased the levels of type 2 cytokines, thereby breaking the vicious cycle of inflammation and barrier damage. Additionally, LTN re-established the expression levels of tight junction protein ZO-1 and repaired the damaged airway epithelial barrier, thereby improving allergic asthma symptoms. The study revealed that both PSAP-1 and PSAP-2 (99), two acidic polysaccharides from Pyrus *sinkiangensis* Yu, significantly increased macrophage vitality, promoted the release of NO, TNF-α, and IL-6, and exerted immunomodulatory effects. Additionally, PSAP-1 particularly improved airway inflammation in asthmatic mice, as manifested by lower expressions of IL-4, IL-5, and IL-13, fewer inflammatory cells, improved lung histopathology, decreased mucus secretion, and resistance to airway inflammation through activation of the NF-κB and MAPK pathways.

Tonifying polysaccharides show promise for multiple sclerosis (MS) prevention and treatment by inhibiting central nervous system (CNS) inflammation through immune cell activation (100, 101). Investigations have demonstrated that *Astragalus* polysaccharides (APS) (102) suppress the proliferation of MOG_35-55_-specific T cells, upregulate the PD-1/PD-L1 pathway, and downregulate the levels of pro-inflammatory cytokines (e. g., IFN-γ, TNF-α, IL-2, IL-17), thereby suppressing Th1- and Th17-mediated immune responses and alleviating experimental autoimmune encephalomyelitis (EAE) symptoms. Acidic polysaccharide of Panax ginseng (APG) (103) significantly retarded the proliferation of PLP_139-151_ peptide-specific T cells, decreased the release of IFN-γ, IL-17, and TNF-α in splenocytes, reduced the number of CD4^+^ T cells in the CNS and CD11b^+^ macrophages, attenuated demyelination and axonal damage, and effectively improved clinical symptoms and histopathological changes in relapsing-remitting experimental autoimmune encephalomyelitis (rr-EAE). The typical etiology of multiple sclerosis (MS) includes central nervous system (CNS) inflammation and myelin destruction. Tonifying polysaccharides may promote myelin repair by augmenting the expression of the anti-inflammatory M2 phenotype. Studies have shown that *Lentinan* (LNT) (104) promotes the phenotypic shift of microglia from M1 to M2 by activating the Dectin-1 receptor, downregulating the expression of the M1 marker iNOS, and upregulating the expression of the M2 marker Arg-1. Meanwhile, LNT also significantly promoted myelin regeneration and motor function recovery in demyelination model mice, thereby demonstrating its potential for treating multiple sclerosis. Rheumatoid arthritis (RA) is a chronic autoimmune disease characterized by immune cell dysfunction and abnormal secretion of pro-inflammatory factors (105). *Angelica Sinensis* polysaccharide (ASP) (106) has demonstrated multiple therapeutic effects in the therapeutic intervention of RA, including marked reduction in joint swelling, inhibiting the release of anti-CII antibodies and pro-inflammatory factors, modulating the gut microbiota by augmenting the proportion of positive anti-inflammatory bacteria (e. g., Lactobacillus) and suppressing the proportion of harmful pro-inflammatory bacteria (e. g., Desulfovibrionaceae), enhancing the production of tight junction proteins (e. g., Cldn5) to repair the gastrointestinal mucosal barrier, and rebalancing the expression of Slit3 and Rgs18 to regulate osteoblast and osteoclast function and reduce bone destruction. *Astragalus* polysaccharide (ASP) (107) significantly reduced toe swelling, inhibited Th17 cell differentiation and the release of pro-inflammatory mediators (e. g., IL-1β, TNF-α), and blocked the NF-κB signaling pathway, thereby alleviating rheumatoid arthritis symptoms in rats. The pathological mechanism of type 1 diabetes mellitus (T1DM) involves the erroneous attack and disruption of pancreatic β-cells by the immune system, resulting in absolute insulin deficiency and subsequent hyperglycemia, and is classified as an autoimmune disease (108). *Lentinan* (LNT) (109) significantly reduced pancreatic islet inflammation scores, decreased β-cell damage, and attenuated pancreatic islet inflammation. Additionally, LNT induces Treg cell proliferation, inhibits the Th1-type immune response, blocks the PI3K/AKT/NF-κB pathway, and demonstrates therapeutic potential for type 1 diabetes mellitus (T1DM) through immunomodulation.

### 4.3 Antiviral infections

Tonifying polysaccharides exert antiviral effects by enhancing T cell regulation, B cell activation and antibody production, macrophage activity, intestinal mucosal immunity, and by directly inhibiting viral replication (110). *Lentinan* (LNT) (111) effectively alleviates diarrhea caused by rotavirus (RV) infection in weaned piglets, with its mechanism strongly correlated with the improvement of the intestinal immune response. LNT markedly elevated serum immunoglobulin expression, including IgA, IgG, and IgM, as well as intestinal secretory IgA (sIgA), strengthened the barrier function of the intestinal mucosal membrane, upregulated TLR3, retinoic acid-inducible gene-I (RIG-I), and melanoma differentiation-associated gene 5 (MDA5) in the intestinal mucosal membrane, and enhanced intestinal immunity. TLR3, retinoic acid-inducible gene-I (RIG-I), and melanoma differentiation-associated gene 5 (MDA5) expression activated the RIG-I/MDA5/MAVS antiviral pathway and promoted the secretion of host defense peptides (HDPs) to enhance intestinal antimicrobial capacity. Refined polysaccharide from *Dendrobium devonianum* (DVP-1) (112) inhibited H1N1 influenza virus infection and attenuated lung inflammation by triggering the TLR4/MyD88/NF-κB pathway, enhancing the release of Th1/Th2-type cytokines, significantly increasing intestinal mucosal IgA expression, and enhancing lymphocyte proliferation and intestinal mucosal immunity. *Sophora flavescens* polysaccharides (SFP-100) (113) reduced Concanavalin A (ConA)-induced hepatocyte necrosis and inflammatory cell infiltration, promoted IFN-γ and IL-6 secretion by splenocytes, and enhanced the Th1-type immune response. Meanwhile, neutral *Sophora flavescens* polysaccharide (SFP-100-A) inhibited hepatitis B surface antigen (HBsAg) and hepatitis B e antigen (HBeAg) in a concentration-dependent manner, with a more pronounced suppressive effect on HBsAg, thereby exerting hepatoprotective and hepatitis B virus (HBV)-inhibitory activities.

Additionally, owing to their natural derivation, minimal toxicity, and absence of residual substances, Tonifying polysaccharides hold potential as vaccine adjuvants in antiviral immunization strategies (114). Polysaccharide from *Ganoderma lucidum* (PS-G) (115), as a mucosal immunopotentiator, significantly enhanced EV-A71 vaccine-induced mucosal (IgA) and systemic (IgG) antibody responses, increased the secretion of IFN-γ and IL-17, and stimulated Th1/Th17-type cellular immunity, thereby providing protection against lethal viral infections. *Astragalus* polysaccharide (ASP) (116), as an adjuvant for inactivated SARS-CoV-2 vaccine (ISV) and recombinant SARS-CoV-2 vaccine (RSV), possesses bi-directional immunomodulatory functions. ASP activates both humoral and cellular immunity by enhancing the release of IgG1 (Th2) and IgG2a (Th1), as well as increasing the expression of cytokines like IL-2, IFN-γ, and IL-4. Meanwhile, ASP adaptively regulates immune responses based on antibody levels: it suppresses excessive inflammatory responses when antibody levels are high and enhances T/B cell proliferation and cytokine secretion when antibody levels are low, thereby maintaining immune homeostasis. *Lentinan*-CaCO_3_ microparticles (CaCO_3_-LNT) (117), as a vaccine adjuvant for H5N1, significantly upregulated the levels of MHC-II and CD86 in lymph node dendritic cells (DCs), promoted DC maturation and activation, elevated the CD4^+^/CD8^+^ T-cell ratio while augmenting the expression of IgG1 and IgG2, thereby activating Th cell immune responses. Studies have shown that TRIM29 and PARP9 are critical regulators of antiviral immunity. TRIM29 deficiency enhances antiviral innate immunity against DNA and RNA viruses, including influenza, by upregulating type I interferon (IFN-I) production in alveolar macrophages (118) and dendritic cells (119, 120). Additionally, TRIM29 deficiency has been shown to mitigate viral myocarditis by modulating PERK-mediated endoplasmic reticulum (ER) stress and immune responses (121). Recent evidence also suggests that TRIM29 deficiency reduces viral enteritis severity through enhanced NLRP6 and NLRP9 inflammasome activation (122). PARP9 is a non-classical RNA sensor that forms a complex with DTX3L (deltex E3 ubiquitin ligase 3L). Within the IFN signaling pathway, PARP9 participates in antiviral responses via ADP-ribosylation. Additionally, PARP9 induces type I IFN production via the PI3K/AKT3-IRF3/IRF7 pathway, thereby playing a pivotal role in antiviral immunity (123, 124). The aforementioned Sophora flavescens polysaccharide (SFP-100) (113), Polysaccharide from Ganoderma lucidum (PS-G) (115), and Astragalus membranaceus polysaccharide (ASP) (116) all promote cytokine production (including IFN-γ) and exhibit antiviral immune activity. Downregulating TRIM29 and upregulating PARP9 also promote IFN-γ production by modulating the IFN signaling pathway, thereby exerting antiviral immune activity. These two mechanisms are highly similar, suggesting that tonic polysaccharides may exert antiviral immune activity through the downregulation of TRIM29 and upregulation of PARP9.

## 5 Link between Tonifying polysaccharide structure and immunomodulation

### 5.1 Extraction and purification methods and structural characterization

Currently, the extraction, separation, purification, and structural analysis of polysaccharides have garnered considerable attention. There are various methods for extracting tonic polysaccharides, each of which significantly affects their molecular weight, monosaccharide composition, glycosidic bond type, and molecular conformation. Common extraction methods include traditional methods (water extraction, acid/alkali extraction, alcohol extraction) and modern auxiliary technologies (ultrasound-assisted extraction, microwave-assisted extraction, enzymatic hydrolysis, etc.) (12, 125). Different extraction methods are suitable for different types of polysaccharides, each with its own advantages and disadvantages (**Table 1**). Extracted polysaccharides often contain impurities such as proteins, water-soluble small molecules, and pigments, necessitating separation and purification. The most commonly used methods for removing proteins include the Sevag method, freeze-thaw method, NaCl method, and CaCl_2_ method. Additionally, ethanol fractionation precipitation and column chromatography are also frequently used purification methods (125). After extraction and purification, polysaccharides can be structurally characterized using various techniques to determine their chemical composition, molecular weight distribution, and glycosidic bond linkage patterns. High-Performance Gel Permeation Chromatography (HPGPC), Fourier Transform Infrared Spectroscopy (FT-IR), Gas Chromatography-Mass Spectrometry (GC-MS), High-Performance Liquid Chromatography (HPLC), Nuclear Magnetic Resonance (NMR), and Ultraviolet-Visible Spectroscopy (UV–Vis) are the most commonly used methods for analyzing the composition and structure of polysaccharides. Structural characterization clarifies the relationship between structure and immunomodulatory activity, guiding the screening and modification of active polysaccharides. The commonly used structural characterization methods for tonic polysaccharides are summarized in **Table 2**.

**Table 1:** Comparison of commonly used methods for extracting polysaccharides.

Extraction method	Extract advantages	Extraction limitations	Applicable polysaccharide types
Hot water extraction method	Easy to operate, low cost	Low efficiency, high impurity content, heat sensitivity	Plant water-soluble polysaccharides (such as *Astragalus* polysaccharides and *Lycium barbarum* polysaccharides)
Acid/alkali extraction method	High efficiency	Structure easily damaged, strict pH control required	Structurally stable polysaccharides (e.g., chitosan, alginic acid)
Alcohol extraction method	Good purification effect	Low extraction efficiency, high cost	Purification of all water-soluble polysaccharides (e.g., *Lycium barbarum* polysaccharides, *lentinan*)
Ultrasonic extraction method	Short time, high efficiency, good activity retention	High cost, polysaccharides easily degraded, temperature control required	Fungal polysaccharides, algal polysaccharides (e.g., *lentinan*, *spirulina* polysaccharides)
Microwave extraction method	Low energy consumption, uniform heating	Polysaccharides easily degraded, temperature control required	Root-derived polysaccharides from plants (e.g., *Notoginseng* polysaccharides, *ginseng* polysaccharides)
Enzymatic hydrolysis method	Mild conditions, few impurities	High cost, long processing time	Thermosensitive polysaccharides (e.g., glycoproteins); plant-derived polysaccharides (e.g., *Polygonatum sibiricum* polysaccharides)

**Table 2:** Common structural characterization methods for polysaccharides.

Structural characterization	Method
Relative molecular weight determination	HPGPC,HPSEC,ELSD,MALLS,RID,HPLC,GPC,MALDI-TOF-MS
Monosaccharide composition analysis	FT-IR,GC,GC–MS,HPLC
Glycosidic bond type analysis	GC,GC–MS,FT-IR,NMR
Conformation and crystal structure	AFM,XRD,SEM,UV,Congo Red Experiment
Structure and functional groups	FT-IR,NMR, GC–MS
Monosaccharide molar ratio	GC–MS,GC,HPLC

The full names of the abbreviations in **Table 2** are as follows: Gel Permeation Chromatography(GPC), Matrix-Assisted Laser Desorption/Ionization Time-of-Flight Mass Spectrometry(MALDI-TOF-MS),High-Performance Size Exclusion Chromatography(HPSEC),High-Performance Gel Permeation Chromatography(HPGPC),Multi-Angle Laser Light Scattering(MALLS),Evaporative Light Scattering Detector(ELSD),Refractive Index Detector(RID),High-Performance Liquid Chromatography(HPLC),Gas Chromatography-Mass Spectrometry(GC–MS),Fourier Transform Infrared Spectroscopy(FT-IR),Nuclear Magnetic Resonance(NMR),UV–Vis Spectroscopy(UV–Vis),Atomic Force Microscope(AFM),X-ray Diffraction(XRD),Ultraviolet Spectroscopy(UV),Scanning Electron Microscope(SEM).

### 5.2 Structure and immunomodulatory activity

The structure of polysaccharides can be categorized into primary and higher-order structures. The primary structure serves as the foundation for the higher-order structure, which represents the specific spatial arrangement of the primary structure (126). Low-molecular-weight polysaccharides (<10 kDa) are mainly linear chains or short branched structures, predominantly distributed in the kidney and intestinal mucosa. These polysaccharides exhibit higher water solubility and stronger tissue barrier penetration capabilities, which enable them to penetrate tissues and exert immune-modulating effects. Medium-molecular-weight polysaccharides (10–100 kDa), which are commonly found in tonic polysaccharides, are primarily distributed in lymph nodes and blood. They exert immune-modulating activity by regulating lymphocytes and dendritic cells. High-molecular-weight polysaccharides (>100 kDa) have complex conformations and are predominantly distributed in the liver and spleen. They cannot easily penetrate the intestinal mucosal barrier and require binding to cell membrane surface receptors to mediate endocytosis or signal transduction, thereby exerting immune-modulating activity. YANG et al. (127) demonstrated that UV/H_2_O_2_ treatment of Moringa oleifera leaf polysaccharides (MOLP) produces the derivative DMOLP-3 (2494 Da). Compared with the original MOLP, DMOLP-3 has a lower molecular weight and broken glycosidic bonds (e.g., a decreased ratio of arabinose to glucose), leading to a simplified structure. This structural change facilitates recognition and fermentation by gut microbiota (such as Bifidobacterium and Bacteroides), thereby producing short-chain fatty acids (SCFAs). SCFAs can inhibit the NF-κB pathway, reduce pro-inflammatory factors (such as IL-6 and TNF-α), enhance intestinal barrier function, and promote Treg cell differentiation, thus balancing Th1/Th2 immune responses. Additionally, the surface morphology of DMOLP-3 changes from a coarse granular to a smooth plate-like structure, with a reduced particle size and enhanced stability. LIANG et al. (48) found that polysaccharides purified from fermented Lentinus edodes polysaccharide (F-LEP-2a) and non-fermented Lentinus edodes polysaccharide (NF-LEP-2a) exhibit distinct characteristics. Fermentation increased the molecular weight of F-LEP-2a from 1.16×10^4^ Da to 1.87×10^4^ Da and altered its monosaccharide composition, with an increased proportion of glucose. This enabled F-LEP-2a to activate macrophage phagocytosis and enhance the recognition ability of immune cell receptors. Compared with the control group, F-LEP-2a increased serum IgG and IgM levels by 42% and 38%, respectively, and outperformed NF-LEP-2a. Therefore, F-LEP-2a exhibits superior immunomodulatory activity. The types and sequence of monosaccharides determine the binding specificity of polysaccharides to immune receptors (e.g., TLR, MR, and Dectin-1). The type of glycosidic bond is crucial for immune activity. Different glycosidic bond structures influence the binding efficiency of immune receptors, signal transduction pathways, and immune cell function, thereby modulating innate and adaptive immune responses. β-glycosidic bonds (e.g., β-1,3, β-1,4, and β-1,6) are key structures for immune activation. Their linear or branched structures readily form helical conformations, enhancing surface receptor binding efficiency and thereby activating the innate immune system. α-glycosidic bonds (e.g., α-1,4 and α-1,6) can generate inhibitory signals to reduce excessive inflammatory responses and are more involved in immune tolerance and adaptive immune regulation. WANG et al. (25) isolated and purified four components (PSP1–PSP4) from Polygonatum sibiricum polysaccharides. Among these, PSP3 is mainly composed of rhamnose (57.69%) and galactose (37.17%). Galactose is linked via β-glycosidic bonds, whereas rhamnose contains methyl protons. These structural features enhance PSP3’s binding capacity to macrophage surface receptors, thereby restoring the Th1/Th2 cytokine imbalance in immunosuppressed mice. ZHANG et al. (73) isolated two heteropolysaccharides, CRPS-1 (193 kDa) and CRPS-2 (9 kDa), from Catharanthus roseus. CRPS-2, a low-molecular-weight polysaccharide primarily composed of galactose and arabinose, exhibits stronger cellular permeability and higher binding affinity with macrophage surface receptors (TLR2/4). CRPS-1 primarily adopts an α-configuration (e.g., α-1,3, α-1,5, etc.), whereas CRPS-2 primarily adopts an α/β-configuration (e.g., α-1,3, β-1,6, etc.), and CRPS-2 has a more complex branched structure, providing more recognition sites. This enables CRPS-2 to more effectively promote the activation of the MAPK/Akt/NF-κB signaling pathway, thereby enhancing NO and IL-6 secretion. In contrast, CRPS-1, due to its high molecular weight and simple branching structure, exhibits relatively weaker structure–activity relationships. Therefore, CRPS-2 demonstrates superior immunomodulatory efficacy. The degree of branching is a crucial structural feature of polysaccharide molecules. Polysaccharides with a moderate degree of branching can provide more receptor binding sites through their highly branched structures, enabling them to bind to multiple receptors on the surface of immune cells, thereby promoting the regulation of immune cells and modulating signaling pathways. However, excessive branching can lead to molecular conformational disorder and excessive steric hindrance, thereby reducing the binding efficiency between polysaccharides and receptors. Additionally, the branching position exhibits specific recognition functionality, with O-6 branching conferring immunological advantages over O-3 branching. FENG et al. (9) found that ginseng polysaccharides, after extraction and purification, yield three fractions: GPS-1a, GPS-1b, and GPS-2. Among these, the simple linear structures of GPS-1a and GPS-1b are less capable of triggering receptor clustering. GPS-2, however, possesses a multi-branched structure and a high aldose content, conferring a stronger binding capacity with the TLR2 receptor. It can activate the TLR2-NF-κB/TRAF6 signaling pathway, thereby significantly enhancing immune responses and repairing radiation damage. Additionally, GPS-2 has a moderate molecular weight (56.86 kDa), facilitating penetration of tissue barriers, while GPS-1a has a high molecular weight (1653 kDa), resulting in lower bioavailability. ZHANG et al. (128) extracted and identified two polysaccharides (CVPn and CVPa) from fruiting bodies of mushroom Coriolus versicolor, both of which have β-1,4 and β-1,3 glycosidic bonds as their main chains. CVPa has a linear main chain with fewer β-1,6 branches, connected via O-6 positions, resulting in stronger binding affinity with Dectin-1 and TLR. Additionally, CVPa has a lower molecular weight (29,783 Da) than CVPn (50,862 Da), making it more effective at promoting receptor dimerization, significantly activating the NF-κB signaling pathway, and inducing iNOS/TNF-α expression compared with CVPn. Therefore, CVPa exhibits significantly stronger immunomodulatory activity than CVPn.

Investigations have demonstrated that some tonifying polysaccharides lack significant immunomodulatory activity on their own, but their immunomodulatory activity is significantly enhanced after structural modification or functionalization (e. g., sulfation, phosphorylation, selenization, carboxymethylation, acetylation). These modifications also alter their properties, including molecular weight, solubility, and thermal stability (129). LONG et al. (130) reported that *Codonopsis pilosula* polysaccharide-modified selenium nanoparticles (CPP-SeNPs) significantly improved immune organ indices, inhibited tumor growth, and increased tumor cell apoptosis in immunosuppressed mice. Furthermore, CPP-SeNPs dose-dependently enhanced the splenic lymphocyte stimulation index, NK cell killing activity, and macrophage phagocytosis activity, and enhanced tumor-specific recognition and killing, thereby showing promise as an adjuvant for anti-tumor immunotherapy. LI et al. (131) extracted polysaccharides from *Marsdenia tenacissima* (Roxb.) and isolated MTP70-1, which has a relative molecular weight of 2738 Da and a main chain primarily composed of (1→6)-linked-D-Glcp and (1→4)-linked-D-Galp. MTP70-Zn was synthesized by introducing zinc into MTP70-1. Both MTP70–1 and MTP70-Zn enhanced macrophage phagocytic activity and promoted the release of cytokines (e. g., TNF-α, IL-6, IL-1β, IL-18) and NO synthesis, with MTP70-Zn showing a more pronounced effect. Additionally, both MTP70–1 and MTP70-Zn influenced TLR4-MyD88-NF-κB pathway-associated proteins by promoting macrophage cytokine expression, with MTP70-Zn exhibiting a higher binding strength for TLR4 proteins. Collectively, MTP70-Zn exhibited stronger immunoreactivity. The corresponding derivatives were obtained by chemically modifying *Atractylodes lancea* (Thunb.) DC polysaccharide (ALP-1) (132) through carboxymethylation (C-ALP-1), phosphorylation (P-ALP-1), and acetylation (A-ALP-1). These derivatives exhibited increased solubility (most pronounced for A-ALP-1), increased molecular weight and thermal stability, and decreased crystallinity. The chemical modifications significantly enhanced the immunomodulatory activity of ALP-1: P-ALP-1 was most effective in inducing NO synthesis by RAW264.7 macrophages, while A-ALP-1 was most effective in promoting TNF-α and IL-6 release. *Poria cocos* polysaccharides (PCP) (133) exhibit poor water solubility. By introducing hydrophilic carboxymethyl groups, two carboxymethyl pachymaran derivatives (CMP-1 and CMP-2) were prepared, which significantly improved their water solubility. Additionally, both CMP-1 and CMP-2 significantly promoted the proliferation and phagocytic capacity of RAW264.7 cells, with CMP-1 exhibiting a significantly greater effect on increasing NO, TNF-α, and IL-6 levels than CMP-2. Following sulfation and hydrolysis modifications of *Polygonatum sibiricum* polysaccharides, over-sulfated (OS) and hydrolyzed (HP) derivatives were obtained. Among these, HP significantly upregulated the expression of iNOS and IL-1β mRNA and activated the MR and TLR4 signaling pathways; whereas OS enhanced the cytotoxic activity of NK cells against HT-29 cells, upregulated the expression of IFN-γ and FasL genes, and promoted their interaction with CR3/TLR2 on the surface of NK cells, thereby enhancing immune regulatory activity (134).

## 6 Conclusion and prospects

The preservation of immune homeostasis is essential for the well-being of the organism. Positive immunomodulatory mechanisms can enhance the host’s ability to resist disease, thereby playing a crucial role in maintaining normal physiological functions. Tonifying polysaccharides can reverse the exogenous suppression of immune organs, including the spleen, thymus, bone marrow, and intestines. Given that immune organs are the core sites for the differentiation and maturation of various immune cells and the initiation of immune responses, tonifying polysaccharides can further regulate the biological activities of lymphocytes, macrophages, dendritic cells, and natural killer cells. These polysaccharides prompt immune cells to generate diverse cytokines and chemokines and exert immunomodulatory effects by regulating signaling pathways, including NF-κB, MAPK, and PI3K/Akt. The immunomodulatory effects of tonifying polysaccharides have broad therapeutic significance, encompassing a variety of diseases, including malignant tumors, inflammatory bowel diseases, rheumatoid arthritis, multiple sclerosis, allergic asthma, type 1 diabetes mellitus, psoriasis, and viral infections. Among them, the role of tonifying polysaccharides as tumor immunomodulators is particularly significant, with mechanisms involving tumor microenvironment remodeling, triggering tumor cell apoptosis, and inhibiting tumor immune evasion (135). **Table 3** summarizes the immunomodulatory effects of polysaccharides.

**Table 3:** Pharmacological effects and mechanisms of tonic polysaccharides.

Name	Models	Method of administration	Dosages	Immunomodulatory actions
GLP-1 (8)	Cyclophosphamide-induced mouse RAW264.7 macrophages	Force-feeding Medium administration	40-160mg/kg 2.5-40μg/mL	Enhance macrophage function by activating the MAPK, PI3K/Akt, and NF-κB signaling pathways through TLR2 and dectin-1 receptor activation.
GPS (9)	Radiated mice RAW264.7 macrophages	Force-feeding Medium administration	50-200mg/kg 100-800μg/mL	Increase the organ index of spleen/heart/liver in irradiated mice, restore bone marrow DNA content, promote macrophage phagocytosis, induce splenic lymphocyte proliferation, upregulate the ratio of CD4^+^/CD8^+^ T cells, and promote the secretion of NO, IL-6, TNF-α, and IL-1β.
PSP3 (25)	Cyclophosphamide-induced mouse RAW264.7 macrophages	Intraperitoneal injection Medium administration	10-400mg/kg 0-400μg/mL	Increase spleen index, promote lymphocyte proliferation, and enhance macrophage and NK cell activity.
LMw-CPP (26)	Cyclophosphamide-induced mouse	Force-feeding	25-100mg/kg	Increase spleen/thymus index, enhance T/B lymphocyte proliferation, NK cell killing activity, macrophage phagocytic capacity, and upregulate IL-2, TNF-α, and IFN-γ levels.
GP1 (27)	Mouse RAW264.7 macrophages	Force-feeding Medium administration	200/800mg/kg 5–5000μg/mL	Increase spleen/liver index, increase T lymphocyte and macrophage counts
FZPS-1 (28)	Cyclophosphamide-induced mouse RAW264.7 macrophages	Force-feeding Medium administration	100/200mg/kg 6.25–100mg/L	Improve immune organ index, carbon particle clearance capacity, enhance macrophage phagocytic capacity, and regulate IL-2 and IFN-γ.
PLP (29)	Cyclophosphamide-induced mouse RAW264.7 macrophages	Force-feeding Medium administration	0.4-1.2g/kg 0.125–1000μg/mL	Restore spleen/thymus index, increase the ratio of CD4+/CD8+ T cells in spleen lymphocytes, and increase CD80/CD86 expression.
PREPS (31)	TNBC mouse	Force-feeding	300mg/kg	Increase the number of HSPCs, LT-HSCs, and CLPs, and reduce the proportion and number of TAMs and MDSCs.
APS (32)	Transwell co-culture	Medium administration	50μg/mL	Upregulates anti-apoptotic proteins Bcl-2 and Bcl-xl, downregulates pro-apoptotic proteins Bax and Bak, alleviates mitochondrial damage and apoptosis, and maintains the proliferation capacity and cell activity of BMSCs.
ABP (34)	RAW264.7 macrophages	Medium administration	2-10μM	Inhibits the RANKL signaling pathway, suppresses osteoclast function, inhibits NFATc1 activity, and MAPK phosphorylation.
CCPs (36)	Rats Caco-2/M-like cells	Force-feeding Medium administration	30/100mg/kg 40–120μg/ml	Downregulation of Claudin-1/ZO-1, enrichment of the Phyllobactalia phylum, activation of TLR4/NF-κB, promotion of IFN-γ/IL-4 secretion, and regulation of Th1/Th2.
HEPs (38)	Mouse RAW264.7 macrophages	Force-feeding Medium administration	500/1000mg/kg 100mg/10mL	Increase gut microbiota diversity, enhance macrophage phagocytic activity, and activate MAPK, PI3K/Akt, and NF-κB signaling pathways.
LDP (39)	S180 sarcoma-bearing mice	Force-feeding	50/100mg/kg	Regulates the intestinal microbiota, promotes adenine-mediated zeatin biosynthesis, alleviates the immunosuppressive microenvironment, upregulates CD4^+^ and CD8^+^ T cell activity, and protects the spleen and thymus.
ABPW1 (41)	Diabetic nephropathy mouse	Force-feeding	300mg/kg	Promotes SCFA production, inhibits histone deacetylases through G protein-coupled receptors (such as FFA2/FFA3), regulates immune cell activity, activates CD4^+^ and CD8^+^ T cells, and promotes M2 macrophage polarization.
ABPs (46)	Cyclophosphamide-induced mice	Force-feeding	50mg/kg	Increase spleen index, promote spleen cell proliferation, increase the proportion of CD3^+^CD4^+^ Th cells in peripheral blood, promote Th1-type immune response, and inhibit Th1-type immune response.
F-LEP-2a/NF-LEP-2a (48)	Cyclophosphamide-induced mice	Force-feeding	100-400mg/kg	Increase spleen/thymus index, boost IgG and IgM levels, promote IL-2 and IL-6 secretion, repair intestinal villi structure, and regulate intestinal flora diversity.
SMEPs (49)	Cyclophosphamide-induced mice	Force-feeding	5-150mg/kg	Increase spleen/thymus index, promote spleen lymphocyte proliferation, enhance macrophage phagocytic capacity, enhance NK cell killing activity, upregulate the proportion of CD3^+^, CD4^+^ T cells and CD19^+^ B cells, and activate Th1/Th2 immune responses.
SYWPP/NYWPP (50)	Cyclophosphamide-induced mice	Force-feeding	6g/kg	Increase spleen/thymus index, elevate IL-2 and IgA levels, increase CD4^+^/CD8^+^ T cell ratio, increase SCFAs content, and improve microbiota diversity.
GLP-3 (52)	RAW264.7 macrophages	Medium administration	50-400μg/mL	Enhances macrophage phagocytic capacity, promotes the secretion of NO, TNF-α, IL-6, IL-1α, IL-1β, IL-10, CXCL5, MIP-2, and MCP-1, and activates the MAPK, PI3K/Akt, and NF-κB signaling pathways.
CPPS-II (53)	Tumor mice RAW264.7 macrophages	Intraperitoneal injection Medium administration	10mg/kg 50-1000μg/mL	Inhibits tumor growth, induces tumor cell apoptosis, increases M1-type macrophages in tumor tissue, elevates TNF-α and IL-6, and reduces IL-10.
PSPs (54)	LPS-induced mouse RAW264.7 macrophages	Force-feeding Medium administration	200mg/kg 5–400μg/mL	Reduce the number of neutrophils and macrophages, upregulate CD206 expression, reduce TNF-α, IL-1β, and IL-6 secretion, reduce M1-type macrophages, increase M2-type macrophages, and inhibit the TLR4-MAPK/NF-κB pathway.
CPPs (56)	RAW264.7 macrophages	Medium administration	6.25–400μg/mL	Promoting macrophage activation by stimulating the TLR4-MAPK/NF-κB pathway
ADP 80-2 (57)	RAW264.7 macrophages	Medium administration	25-100μg/mL;	Enhance macrophage phagocytic capacity and increase IL-6, IL-1β, TNF-α, and NO secretion.
*Angelica gigas* Polysaccharides (60)	RAW264.7 macrophages NK-92 natural killer cells HCT-116 cells	Medium administration Medium administration Medium administration	75-300μg/mL 75-300μg/mL 75-300μg/mL	Promotes macrophage secretion of NO, TNF-α, IL-1β, and IL-6, induces NK-92 expression of IFN-γ, TNF-α, NKp44, and Granzyme-B, enhances cytotoxicity against HCT-116, and activates the NF-κB and MAPK pathways.
*Cnidium officinale* polysaccharides (61)	RAW264.7 macrophages NK-92 natural killer cells HCT-116 cells	Medium administration Medium administration Medium administration	5–100μg/mL 5–100μg/mL 5–100μg/mL	Promotes NO production, upregulates the expression of genes such as iNOS, TNF-α, IL-1β, IL-6, and IL-10, enhances the cytotoxicity of NK cells against HCT-116, and activates the NF-κB and MAPKs pathways through CR3 receptor activation.
IRPS (64)	MoDCs cells	Medium administration	3.125μg/mL	Promotes DC maturation, upregulates IL-10, IL-1β, and IL-12p38 mRNA levels, and inhibits PRV.
PEI-PSP-CaCO3 (65)	Mouse	Subcutaneous injection	200μL/只	Significantly elevated IgG levels, activation of dendritic cells, and promotion of T cell activation, activating humoral/cellular immunity.
LBPL (66)	Mouse bone marrow-derived DCs	Medium administration	31.25–125μg/mL	Promotes DC maturation through the TLR4-MyD88-NF-κB signaling pathway, enhancing antigen presentation and Th1 cytokine secretion.
sCAP (67)	Mouse bone marrow-derived DCs	Medium administration	0.49–7.81μg/mL	By activating the MyD88/NF-κB signaling pathway, it promotes DC maturation and Th1 cytokine secretion while inhibiting excessive inflammatory responses, thereby exerting both immune-enhancing and anti-inflammatory effects.
BCAP-1 (69)	S-180 sarcoma mouse	Force-feeding	100–400mg/kg	Activate macrophages through the NF-κB signaling pathway to promote the secretion of cytokines such as TNF-α and NO.
sHEP (70)	Mouse bone marrow-derived DCs	Medium administration	0.781-12.5μg/mL	Promotes DC maturation and Th1 cytokine secretion, through TLR4 activation of MAPK and NF-κB pathways.
CRPS-2 (73)	RAW264.7 macrophages	Medium administration	0.16–5μg/mL	Activation of the MAPK/Akt/NF-κB pathway through TLR2/TLR4 receptors promotes macrophage proliferation, phagocytosis, and inflammatory factor secretion.
LGP (78)	Canavanine-induced mice Canavanine-induced RAW264.7 cells	Force-feeding Medium administration	100/200mg/kg 0-800μg/ml	By inhibiting the PI3K/AKT and TLRs/NF-κB signaling pathways, reducing the secretion of inflammatory factors and inhibiting hepatocyte apoptosis, thereby alleviating the inflammatory response in autoimmune hepatitis (AIH).

Although numerous studies have investigated the immunomodulatory effects of tonifying polysaccharides, several deficiencies remain. (1) Current studies on the immunomodulatory activities and mechanisms of tonifying polysaccharides primarily focus on basic immunology, with immune organs, immune cells, and immune factors serving as the primary benchmarks. However, there is a notable absence of studies integrating multi-omics technologies, such as metabolomics, genomics, and transcriptomics, to systematically assess the effects of these polysaccharides on the organism’s immune functions. Additionally, research integrating metabolomics, genomics, transcriptomics, and other multi-omics techniques to systematically evaluate their impact on immune function is lacking. Consequently, the existing studies have not fully and systematically elucidated the specific mechanisms through which tonifying polysaccharides influence immune activity. (2) At present, investigations on the immunomodulatory effects of tonifying polysaccharides and their mechanisms primarily rely on animal models and *in vitro* cytological analyses, with a marked deficiency in clinical research data. Given this, systematic clinical trials are necessary to evaluate the safety and efficacy of tonifying polysaccharides in human applications. (3) The immunomodulatory therapeutic effects of tonifying polysaccharides are extensive and have garnered significant attention in current academic research. However, research on combining tonifying polysaccharides with other drugs to achieve additive or synergistic effects remains limited and requires further in-depth exploration. The combination strategy involving tonifying polysaccharides can not only augment efficacy and reduce side effects but also offer a crucial direction for the prophylaxis and therapeutic intervention of immune-mediated disorders. This strategy has opened up broad prospects for the clinical application of tonifying polysaccharides and the development of novel immunomodulatory drugs. (4) The complex structure of polysaccharides in tonic agents poses challenges for quality control, as most rely on total sugar content as an indicator, leading to significant variations in therapeutic efficacy. It is necessary to clarify the structural parameters of polysaccharides (molecular weight, monosaccharide composition, branching degree, glycosidic bond type), integrate structural characterization techniques (e.g., HPLC, FT-IR, GC–MS, etc.) with immune function evaluation, and establish a dynamic correlation model linking “extraction method-structural characteristics-immune activity” to provide a scientific basis for the standardized production and precise application of tonic polysaccharides. (5) There is insufficient investigation into the advanced structures of tonifying polysaccharides (e. g., helical structure, electric charge characteristics, spatial aggregation state). Nuclear magnetic resonance (NMR), atomic force microscopy (AFM), and other techniques can be employed to precisely elucidate the relationship between polysaccharide immunoregulatory activity and spatial conformation. (6) The pharmacological effects of tonifying polysaccharides are primarily realized through oral administration, yet their inherently low bioavailability limits the full realization of their efficacy. Their immunomodulatory activity can be significantly enhanced through structural modification strategies, such as acetylation, sulfation, phosphorylation, and selenization. However, research on structural modifications aimed at improving the oral absorption efficiency of tonifying polysaccharides remains limited. In-depth studies on structural modifications are of great scientific significance and application value for enhancing the immunomodulatory efficacy of tonifying polysaccharides and promoting the creation of novel immunomodulators. (7) Owing to their complex composition, individual differences, and improper use, polysaccharides in tonic supplements may cause side effects, including immune overreaction, allergic reactions, and gastrointestinal disturbances. Therefore, strict dosage control is necessary during use. Patients with autoimmune diseases should avoid using them. Injectable polysaccharides must undergo protein residue testing, and patients should be asked about their allergy history before administration. It is recommended to start with a low dose and combine with probiotics to regulate the intestinal flora. Future studies with larger sample sizes are required to further elucidate their safety profiles. (8) Although the antiviral immune effects of tonic herbs share similarities with the role of the TRIM29/PARP9 in antiviral immunity, there is currently a lack of direct evidence demonstrating that tonic herbs exert their antiviral immune effects through the regulation of TRIM29/PARP9. Future studies should further investigate the interaction mechanisms between polysaccharides and the TRIM29/PARP9 to provide theoretical support for the development of antiviral drugs based on polysaccharides.
